# Validation of the metacognitive skills questionnaire for drivers of vehicles (CHMC)

**DOI:** 10.3389/fpsyg.2023.1054279

**Published:** 2023-02-28

**Authors:** Jose Luis Antoñanzas, Carlos Salavera

**Affiliations:** Departament of Psychology and Sociology, Universidad de Zaragoza, Zaragoza, Spain

**Keywords:** metacognitive, skills, planning, self-regulation, evaluation

## Abstract

**Introduction:**

Knowing what strategies users use in the difficult task of driving has always been a priority objective in road safety, given that road accidents are one of the main causes of death in the world, as confirmed by the WHO. In this sense, the metacognitive skills questionnaire for drivers was validated.

**Methods:**

The questionnaire measures the metacongitive skills used by vehicle drivers at three times before, during and after driving.

**Results:**

The results of both the exploratory factor analysis (0.92 alpha by Cronbach) and the confirmatory factor analysis show the existence of three factors, a planning factor, a self-realization factor, and a third evaluation factor.

**Discussion:**

Finding these results together with a psychoeducational intervention design, will improve the behavior of drivers and in turn will serve to improve the training programs of the same to the different institutions and centers responsible for such training.

## 1. Introduction

Traffic accidents have, in the last decade, been one of the main causes of mortality all over the world. According to WHO data, more than 1.2 million people died in traffic accidents in 2021. The latest data about such accidents show that they are now the tenth cause of death worldwide. According to the indicator “*years of potential healthy life lost,”* which the WHO employs, accidents are the ninth cause of life hazards (Mirón, and Laborín, 2015). There is absolutely no doubt that traffic accidents are a serious problem for health, and also for the economy. Needless to say, it is a complex phenomenon that results from a series of factors linked to the environment, the vehicle itself and to human beings (Alonso et al., 2021a). The scope of this problem, and the fact that it persists over time, have led to different disciplines being placed in charge of investigating it, and one of the most important is Psychology because it studies the human factor. It is necessary to bear in mind that conducts which put driving at risk represent more than 5% of causes of accidents (McKnight and McKnight, 2003; Ivers et al., 2009; Siliquini et al., 2011). Thus, inappropriate and risky behavior by road users is the main cause of road accidents (Siliquini et al., 2010; Montes et al., 2014; Valero-Mora et al., 2021). For this reason, several preventive measures have been developed to change the attitudes and behavior of road users, such as educational programmes and communication campaigns (Faus et al., 2021; Alonso et al., 2021b).

Evidently, driving a car is no simple task and can, as previously mentioned, be considered one of the riskiest conducts for human health (Antoñanzas et al., 2015). One widely accepted definition today from the cognitive psychology perspective is that it entails the complex task of controlling a constantly changing mechanism in movement in an environment with many factors, and performing subtasks at the same time, such as controlling one’s route or changing gears. This is the reason why different aspects of driving (attention, perception, memory, or motivation) have been studied, as have matters related with personality, anxiety and emotional factors. They all share a common objective: to minimize risky conducts or behaviors while driving (Evans and Carrère, 1991; Castro et al., 2006; Sommer et al., 2008; Bucchi et al., 2012; Richards and Charlton, 2020).

We should also take into account drivers’ limited capacity to process information about all the factors that intervene in traffic. Driving becomes an activity that involves selecting stimuli and decision making. Indeed driving has been defined by authors working in the ergonomics field and those who study human factors as an *automatic self-regulated* task (Brown, 1980; Anderson, 1993). Others like Tudela (1992) use driving to illustrate the differences between controlled processing and the creation of automatisms by linking the differences between two processes with stages in which certain cognitive or motor learning is acquired. The first stages of learning are dominated by controlled processing, while automatic processes lead this activity after considerably practicing the task.

Although, generally speaking and with time, driving has been considered an automatic-type skill because it is frequently and systematically carried out (Anderson, 1993), this is not altogether clear as many authors have their doubts about it being an automatic activity. Whereas cognitive psychologists have always considered the task of driving to be automatic, several research works have shown that driving efficacy diminishes when performing other tasks while driving, which demonstrates that it is not a completely automatic task (Groeger, 2000; Love et al., 2022).

Thus assessing drivers with psychometric tests is absolutely necessary, especially for professional drivers, as some recent research works have indicated (Vetter et al., 2017). Several countries traditionally set psycho-technical tests to assess their drivers’ performance (Austria, Spain, Hungary, etc.). These tests are used in the medical-psychological exams used to evaluate drivers’ cognitive requirements. However, we stress that the theoretical precepts underlying these tests have remained virtually unaltered to date, whereas the new technological advances in the automobile industry and transport have vastly changed (Alonso et al., 2009; Sayed et al., 2010; American Trucking Association, 2016).

Assessing drivers’ cognitive capacities is a fundamental part to evaluate their performance, as previously indicated. Of these, metacognition is a basic strategy to learn about the cognition and its regulation. According to several authors’ definition of metacognitive activity as the capacity to handle cognitive resources, i.e., knowledge about cognitive processes and regulating these processes (Wellman, 1985; Brown, 1987; Martí, 1999), metacognitive activity is composed of planning, supervision and evaluation processes. Metacognitive skills facilitate and improve learning. According to Taylor (1983), metacognitive skills are understood as knowledge of a task, the possible strategies that can be applied to a task, and the individual conscience of skills themselves in relation to these strategies.

Driving involves *regulating* the processing of driving by paying attention and releasing resources when a task becomes automatic. When people start learning to drive, they need to pay attention to every component involved in this skill. With practice, mastering these components becomes more fluent and performing each component requires drivers paying less attention, which spells more relaxed and calmer driving (Castro et al., 2006). When performing the various subtasks that driving implies, a cost comes into play, which can be reduced only if drivers are informed in advance about what they will do (*via* cognitive signs or strategies).

## 2. Objectives and hypothesis

The main objective of this work was to describe the validation of factor measures related to the metacognitive processes or skills used by subjects in the driving task using the metacognitive skills questionnaire for drivers, CHMC. It is hypothesized that, given the proven reliability, consistency and validity of the Metacognitive Skills for Drivers Questionnaire, and its adaptability to different driving situations, before, during and after it, the confirmatory model based on three factors following the instrument will present a good fit and optimum factor loads. It should also be noted that there may be some variations of factors in what is the structure of the instrument, especially if you consider the experience or professionalism of the driver. In this sense it is hypothesized that those professional drivers or with more hours or km traveled have greater metacognitive skills than novice drivers or with less experience in driving.

## 3. Materials and methods

### 3.1. Sample

A sample with 570 drivers aged between 18 and 63 years was recruited. The percentages of males and females were 56 and 44%, respectively. In age terms, below shows that the highest percentage of drivers fell in the 36-50-year-old age group (57%), and finally by the group with drivers aged more than 50 years (18%) (*X* = 41.79; SD = 9.13). Their driving amounted to *X* = 12.77 years; SD = 12.87, and the mean number of kilometers driven a year was 11,000 km/year.

### 3.2. Study design and procedure

A descriptive cross-sectional study was performed. Users answered a metacognitive skills questionnaire along with another series of personality, anxiety and emotional intelligence questionnaires. Drivers were selected through a convenience (non-probability) sampling method. The process for passing such tests took more than 6 months.

All the participants received a series of guidelines to complete the questionnaire. For all the tests, they were informed about the study and their anonymity was guaranteed. They all participated voluntarily. It is made with the participation of professional drivers belonging to different groups (Taxi, freight transport, passenger transport, ambulances, delivery, and commercial), and novice drivers. Sample mortality was 12%.

## 4. Description of the questionnaire

### 4.1. The metacognitive skills questionnaire for drivers of vehicles

This questionnaire is used to check the different types of metacognitive skills that a subject uses when driving a vehicle. The questionnaire is divided into three clearly different parts. Regulating and controlling knowledge refer to a *driver’s active participation* at three time points: before the driving activity commences (predicting, organizing, etc.,), during the driving process (adjusting, revising, etc.,) and after driving (evaluating, feedback, etc.,).

The subjects had to answer a series of statements they were presented with, which responded to the principles of metacognitive skills (Brown, 1987). Each item is scored from 1 to 6, where 1 is always and 6 is never.

*Controlling cognitive processes:*

*Planning*: designing the steps to take.

For example,: you plan the journey, *I know which way to go*, etc.

1. *Self-regulation*: follow each planned step.

For example: *I control what I am going to do before I start driving*

1. *Evaluation*: Evaluate each step both individually and as a whole.

For example: *I evaluate the situation in detail.*

1st PART. BEFORE USING THE VEHICLE.

This part asks about everything the subject does before starting a journey, from finely tuning the vehicle to planning the journey. It is a matter of looking at the planning a driver does before starting to drive:

**Table:** 

I know which way to go.
I plan a long route before starting to drive.
I control what I am going to do before starting to drive.
I think about my physical condition (discomfort, medicines, etc.).
I think about my psychic condition (stress, aggressiveness, etc.).
I mentally solve problems.
I think about not making mistakes.
I think about not breaking rules.

2nd PART. DURING THE DRIVING PROCESS.

This checks the different self-regulation mechanisms that a driver uses when driving, from anticipating own conducts to visualizing possible risks.

**Table:** 

I think about how I am driving.
I think about similar situations.
I evaluate the situation in detail.
I know and control distractions.
I think about my decisions.
I remember the rules.
I try to control impulses.

3rd PART. AFTER DRIVING.

After driving the route, the individuals were asked to analyze what they had experienced. Checks were made to see if they were capable of correcting mistakes or solving problems. It was a matter of them evaluating their driving by following a process by which to reflect on and analyze the task of driving:

**Table:** 

I go over and see if I have made any mistakes.
I remember how I have driven.
I evaluate situations in detail.
I mentally solve problems.
I go over other people’s mistakes.
I plan taking future actions.

### 4.2. Ethics statement

For this research, the Ethics Research Committee of OPIICS (S46_20R), Department of Psychology and Sociology, University of Zaragoza was consulted. In addition, the study responds to the general ethical principles necessary for research in Social Sciences, and its conformity with the Declaration of Helsinki was certified. Study participants read and signed an Informed Consent Statement they received before completing the questionnaire. It contained ethical principles and details of data processing, specifying the purpose of the study, approximate duration of the questionnaire, processing of personal data and explaining voluntary participation, as well as the right to withdraw from the investigation at any time. The research in order to avoid any potential risk to the integrity of the collaborators of the study, did not use personal and/or confidential data, being anonymous participation.

### 4.3. Data processing (statistical analysis)

First, descriptive analyses (averages, standard deviations and other basic measurements) of the sample were performed. Subsequently, an analysis of the factorial structure of the CMHC was performed, first by means of an exploratory factor analysis (AFE) with the maximum likelihood method, to discover the underlying structure of the set of variables. Model adjustment indices and reliability/consistency scores were considered using Cronbach’s alpha. Later, confirmatory factor analysis (CFA) was evaluated with the SPSS AMOS program (version 24.0). The adjustment of the model was evaluated by several statisticians (Chi-square, GFI, CFI, NFI, TLI, and RMSEA) as recommended in the literature. The adjustment was established based on the cut-off criteria suggested by Marsh et al. (2004): a CFI greater than 0.90 (better if greater than 0.95) and an RMSEA lower than 0.08 indicates an appropriate fit of the model.

## 5. Results

### 5.1. Validation of the CHMC

The first step was to study the purpose of the metacognitive skills questionnaire for drivers (CHMC). To do so, the statistical data offered in Table 1 were acquired. This analysis informed us about the number of elements (variables) included in the analysis, and also about the reliability coefficient value (Cronbach’s alpha). From the scale’s reliability point of view, this coefficient was excellent (0.909; see Table 1), where values over 0.8 are considered good, and those over 0.9 are excellent (Pérez-Gil et al., 2000). The CHMC values are high, which indicates excellent internal consistency among the scale’s factors.

**Table 1:** The reliability coefficients of the CHMC scale.

	Cronbach’s alpha	Cronbach’s alpha based on typified elements	No. elements
CHMC	0.909	0.911	21

After finishing this first stage, the next stage consisted in running a factor analysis of the scale (Table 2). This analysis is a technique which, by reducing data, is used to explain the variability among the observed variables in terms of fewer non observed variables called factors. Saturated values <0.02 (in absolute values) were eliminated. As very small saturations tend to have no interpretable value, eliminating them from the table allows attention to be paid more easily to more relevant saturations.

**Table 2:** Factor analysis.

		1	2	3
I know which way to go	a1			0.431
I plan a long route before starting to drive	a2			0.372
I control what I am going to do before starting to drive	a3			0.474
I think about my physical condition	a4			0.846
I think about my psychic condition	a5			0.827
I mentally solve problems	a6			0.475
I think about not making mistakes	a7			0.673
I think about not breaking rules	a8			0.573
I think about how I am driving	dr 9	0.582		
I think about similar situations	dr10	0.468		
I evaluate the situation in detail	dr11	0.671		
I know and control distractions	dr12	0.631		
I think about my decisions	dr13	0.752		
I remember the rules	dr14	0.761		
I try to control impulses	dr15	0.642		
I go over and see if I have made any mistakes.	d16		0.843	
I remember how I have driven	d17		0.750	
I evaluate situations in detail	d18		0.853	
I mentally solve problems	d19		0.676	
I go over other people’s mistakes.	d 20		0.626	
I plan taking future actions.	d 21		0.679	

## 6. Rotation method: Varimax Kaiser’s normalization

### 6.1. Rotation converged in five iterations

Three clearly different factors exist. The first one (see Table 2) would be saturated by the variables that come into play before starting to drive: I know which way to go (0.474), I control what I am going to do (0.479), I plan a long route (0.372), I think about my physical condition (0.846) and about my psychic condition (0.827), I think about not making mistakes: these are all variables that can be used to plan. A second factor would be explained by the skills that come into play when driving: I evaluate the situation in detail (0.671), I think about my decisions (0.752), I remember the rules (0.761), I know and control distractions (0.631), I try to control impulses (0.642), I think about how I am driving (0.582). The third and final factor consists in the skills that come into play when an individual has stopped driving: I go over and see if I have made any mistakes (0.843), I evaluate situations in detail (0.853), I remember how I have driven (0.750), I plan future actions (0.679), I go over other people’s mistakes (0.626) and I mentally solve problems (0.676).

In general, high values were obtained for each factor. Therefore, three factors exist which generally coincide with the three time points while performing the driving task: before, during and after. After performing the exploratory factor analysis, confirmation was provided with the factors structured by the SEM method to demonstrate the procedures and logics of this statistical tool, and to also make recommendations to improve the theory, as well as the instrument used to measure drivers’ learning capacities. Nowadays research uses such multivariate methods to adequately understand the complexity of psychological phenomena. We ought to bear in mind that the volume of multivariate techniques used in social sciences, particularly in psychology, is quite substantial. Certain techniques stand out, like multiple regression or factor analyses. Structural Equation Modeling (SEM) allows simultaneous examinations to be made of a series of dependence relationships, which is very useful when a dependent variable becomes an independent variable in ulterior dependent relationships (Cupani, 2012). It can be stated that structural equations are a kind of conglomerate made up of a mixture of several techniques, such as multiple regression and factor analyses (Kahn, 2006).

**Figure 1:**
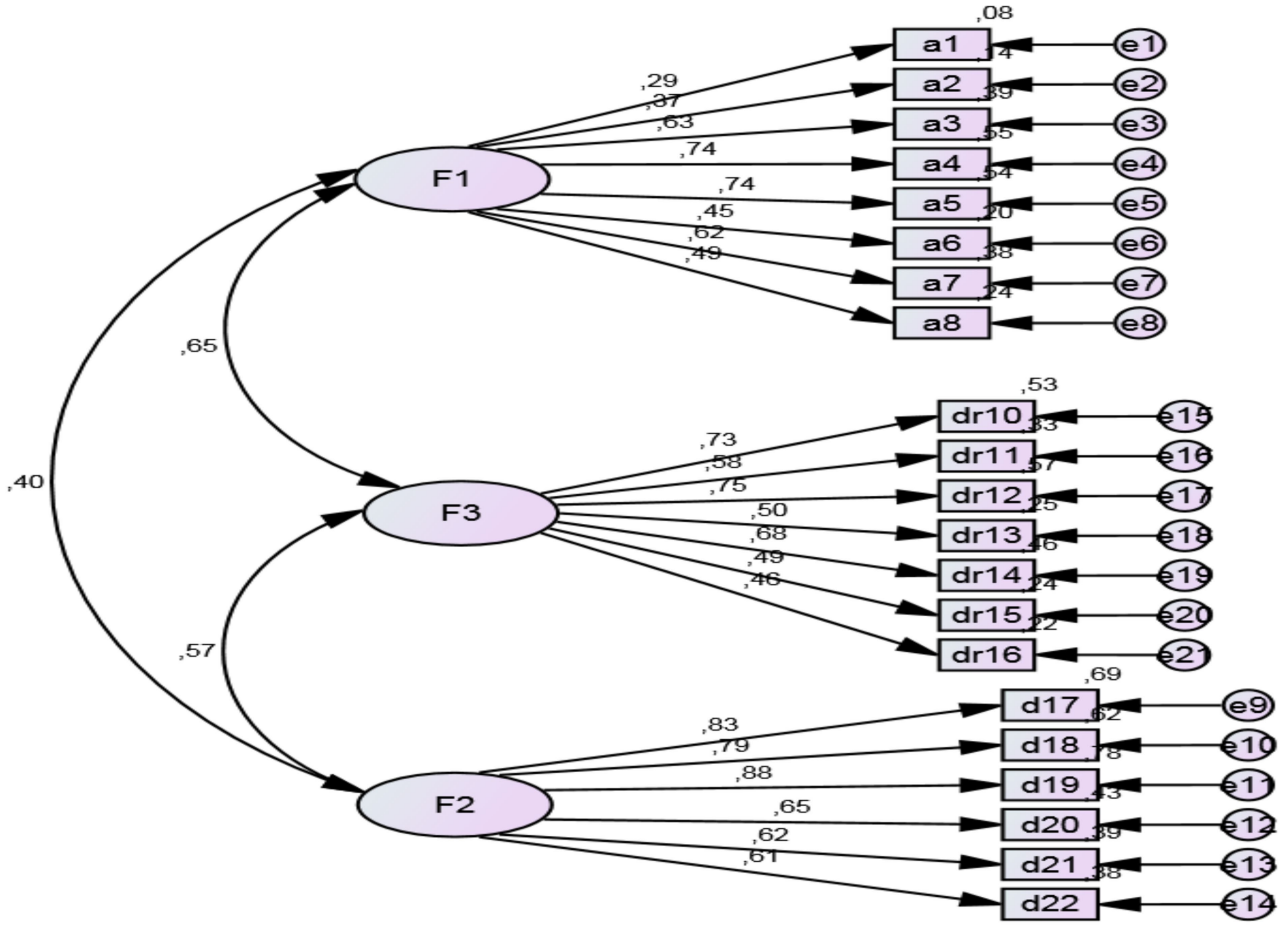
The structural equations of the CHMC.

In Figure 1, the latent variables represented with ellipses correspond to Factor 1, Serious Planning (before driving), Factor 2, Self-regulation (during the driving process) and Factor 3, Evaluation (after driving). The measured variables (indicators) are represented by rectangles. Each measure or latent variable can be exogenous (independent) or endogenous (dependent). All the indicators in Figure 1 (e.g., F1, F2, etc.,) are endogenous as they are dependent (predicted) by their respective latent variables. One of the fundamental matters with SEM is that dependent variables tend to have variation that is not explained by the latent variable, and is attributable to the measuring error. Thus any variance of the error must be modeled. Figure 1 offers the confirmatory factor analysis (CFA) result, along with the structural equations of the method that obtained the maximum likelihood. This confirmed that the model was suitable because a sustainable model was achieved. The normalized regression coefficients were statistically significant (*p* < 0.05), with values over 0.5, so all the indicators were satisfactorily saturated with the latent variable.

As the different fit indices were suitable for the model’s fit, it can be stated that the model proposed for the factor scale structure was sustainable: *χ*^2^ (189) = 439.614; *p* = 0.001; *χ*^2^/gl = 2.326; GFI = 0.94; AGFI = 0.96; CFI = 0.96; NFI = 0.94; TLI = 0.93; RMSEA = 0.069, 90% CI (0.051–0.112).

In short, our results revealed that, in principle, the relationships between the considered dimensions and the observed variables obtained the expected congruence. This means that positive and negative relationships exist between the two elements as theory postulates.

## 7. Discussion

This cross-sectional empirical work had as its main objective to present the validation of the Metacognitive Skills Questionnaire for Drivers (Antoñanzas et al., 2015). It can be said that the results obtained in this research confirmed that, both in the exploratory evaluation and with the theoretical and empirical references on which the CHMC is based, the questionnaire maintains a well-adjusted configuration of three factors, and guarantees a good method for measuring metacognitive skills in driving, which means for the implementation of prevention and driver training programs.

In this sense we must remember in a definition of metacognition, Crespo (2000) analyzed the various controversies that arose and coined the term metacognition. Weinert (1987) argues that metacognition is, in a general sense (cognition over cognition), a conceptually clear term, but loses its clarity when applied to specific situations, although as this study has shown drivers before, during and after driving, It is a metacognitive type activity. For Jacobs and Paris (1987), there are some aspects of metacognition for which there is no agreement, where the positions of the different researchers are irreconcilable: for example, affective factors; for example, Flavell (1979) states that they are present in the construct (“metacognitive experience”), but Brown (1985) seems to take them as a single cognitive element. The research showed that there is a greater use of metacognitive skills by drivers who have more experience in driving, which is consistent with Flavell’s position. However, one of the main differences lies in the level of awareness of what is metacognitive: whether knowledge and the deliberate actions of a subject, or whether those that are tacit and automatic in nature, can be considered metacognitive processes. The results of this work seem to suggest the latter. The implementation by drivers of metacognitive skills seems to be a reality according to the data obtained, not only those that are seen but also those that exist tacitly, case of preparation for driving or after.

The existence of metacognitive skills in driving, as is being commented, has been one of the objectives of the research, hence the realization of a questionnaire to measure these abilities, it is therefore important to remember the existing literature on metacognition as well, for authors like Nisbet and Shucksmith (1987), metacognitive skills control and regulate the skills that correspond to tasks or practices. On the one hand, they refer to an individual’s conscience and knowledge of his/her own cognitive processes; *knowledge of knowledge*; on the other hand, a *control capacity* of these processes exists as they can be organized, managed and amended to fulfill learning goals (Flavell, 1976, 1977; Allueva, 2002). According to Monereo (1997), they are *macrostrategies* as they are not specific strategies, but general ones, that take a mental direction, so their degree of transfer is higher (Colombo et al., 2007). In line with the driving task, current research works have advanced in cognitive processes to explain differences in drivers’ conducts (Hu et al., 2013). For example, there are a series of variables, like age, gender and experience, as well as cognitive variables, like mistaken judgment, which are related with driving mistakes (Taubman-Ben-Ari et al., 2004; Wiesenthal and Singhal, 2006; Bjureberg and Gross, 2021). Failures in the cognitive procedure are associated with lack of confidence and the possibility of having an accident being more likely, as some recent research works have recently demonstrated (Chai et al., 2017). Hence, the importance of being able to measure the metacognive processes that drive drivers.

A relationship with cognitive processes and metacognitive skills was verified (planning, self-regulation, evaluation), as Brown (1987) defined, using the items or variables in the Metacognitive Skills Questionnaire for Drivers of Vehicles. As part of metacognition development, there are two ways of interpreting it. Some authors defend that age is a determining variable because drivers’ regulation process levels increase as they grow older (Flavell, 1979; Brown, 1987; Karmiloff-Smith, 1995). Others like Pozo (1996) stress that skill is the determining factor because the subject’s cognitive and self-regulating capacity increases. There is no doubt that age is a determining factor in driving because older drivers employ more metacognitive skills, and the more kilometers driven, the more drivers use self-regulation processes. Sternberg (1983) clearly differentiates between executive and non executive skills: the former allow us to plan, control and revise the strategies we adopt to perform a task, while the latter are specifically employed to perform a task (Clavero, 2001). The CHMC clearly reflects this acquisition and development of metacognitive skills as the oldest drivers with the most driving experience are those who obtained higher scores for the three factors and/or driving time points.

In line with the results obtained by the factor analysis done of the CHMC, we found that the scale’s skills matched these results, and in such a way that the resulting three factors in the analysis corresponded with the metacognitive activities according to the above-cited authors. The following were found: a planning factor composed mainly of those skills that take place before starting to drive; a second supervision factor made up of the items used during the driving process; a third evaluation factor that comprises the skills that come into play when driving has ended. Hence the scale fits the main theories about metacognitive activity by confirming its objective: that of measuring drivers’ metacognitive skills at three driving time points. Recent studies have confirmed that an overall evaluation using psychometric tests is recommended to evaluate safe driving conduct (Vetter et al., 2017; Tice et al., 2022).

## 8. Conclusion

It can be concluded that the results obtained in this work support the hypothesis that the CHMC serves to measure the cognitive skills that users use in the task of driving and therefore is useful for improving road safety. Greater use of metacognitive skills when driving helps minimize risks on the tracks. All this can be used to carry out driver training programs, both new and professional in relation to metacognitive factors or strategies.

### 8.1. Limitations of the study

Although the sample is representative and sufficient, and the statisticians used are adequate in this type of empirical studies, this research has a number of limitations. The sample could be extended by population sectors, that is, by classes of drivers, habitual, unusual, car, motorcycle, etc. Carry out a study with a greater variety of samples. On the other hand, surveys with questionnaires often have problems of bias in the answers, Expanding to a type of questionnaire with open or semi-structured questions could give a more accurate or tighter view of the type of metacogitive skills that users use on the roads (Useche et al., 2019). The use of other types of regression statistics, such as mediational analysis, would allow us to consider other possible variables that are influencing the use of metacognitive skills in driving.

## Data availability statement

The original contributions presented in the study are included in the article/supplementary material, further inquiries can be directed to the corresponding author.

## Ethics statement

Ethical review and approval was not required for the study on human participants in accordance with the local legislation and institutional requirements. Written informed consent from the [patients/ participants OR patients/participants legal guardian/next of kin] was not required to participate in this study in accordance with the national legislation and the institutional requirements.

## Author contributions

All authors listed have made a substantial, direct, and intellectual contribution to the work and approved it for publication.

## Conflict of interest

The authors declare that the research was conducted in the absence of any commercial or financial relationships that could be construed as a potential conflict of interest.

## Publisher’s note

All claims expressed in this article are solely those of the authors and do not necessarily represent those of their affiliated organizations, or those of the publisher, the editors and the reviewers. Any product that may be evaluated in this article, or claim that may be made by its manufacturer, is not guaranteed or endorsed by the publisher.
